# The Experience of Testing for Coronavirus Disease (COVID-19) at a Single Diagnostic Center in Paraguay before the Introduction of Vaccination

**DOI:** 10.3390/v15051136

**Published:** 2023-05-10

**Authors:** Florencia del-Puerto, Leticia E. Rojas, Chyntia C. Díaz Acosta, Laura X. Franco, Fátima Cardozo, María E. Galeano, Adriana Valenzuela, Alejandra Rojas, Magaly Martínez, Ana Ayala-Lugo, Laura Mendoza, Fátima S. Ovando, Mario F. Martínez, Hyun J. Chung, Richard Webby, Eva Nara, Miguela A. Caniza

**Affiliations:** 1 COVID-19 Contingency Technical Committee of the Instituto de Investigaciones en Ciencias de la Salud (IICS) of the Universidad Nacional de Asunción (UNA), San Lorenzo 111241, Paraguay; colepuerto@gmail.com (F.d.-P.); letyroj@hotmail.com (L.E.R.); chyntiacarolinadiaz@gmail.com (C.C.D.A.); laurafpy@hotmail.com (L.X.F.); fati.cardozo@hotmail.com (F.C.); maruphd@hotmail.com (M.E.G.); abvalenzuela80@gmail.com (A.V.); alerojaspy@gmail.com (A.R.); magaly.martinez@gmail.com (M.M.); anaayalalugo@gmail.com (A.A.-L.); lauramendozatorres@gmail.com (L.M.); mfmarmora@gmail.com (M.F.M.); 2 Departamento de Biología Molecular y Biotecnología, Instituto de Investigaciones en Ciencias de la Salud, Universidad Nacional de Asunción, San Lorenzo 111241, Paraguay; 3 Departamento de Salud Pública, Instituto de Investigaciones en Ciencias de la Salud, Universidad Nacional de Asunción, San Lorenzo 111241, Paraguay; 4 Departamento de Producción, Instituto de Investigaciones en Ciencias de la Salud, Universidad Nacional de Asunción, Dr. Cecilio Báez y Dr. Villamayor, Campus Universitario, San Lorenzo 111241, Paraguay; 5 Departamento de Genética, Instituto de Investigaciones en Ciencias de la Salud, Universidad Nacional de Asunción, Dr. Cecilio Báez y Dr. Villamayor, Campus Universitario, San Lorenzo 111241, Paraguay; 6 Departamento de Control de Infecciones, Facultad de Ciencias Médicas, Hospital de Clínicas, Universidad Nacional de Asunción, Campus Universitario, San Lorenzo 111241, Paraguay; fatima.ovando@gmail.com; 7 Dirección General, Instituto de Investigaciones en Ciencias de la Salud, Universidad Nacional de Asunción, Dr. Cecilio Báez y Dr. Villamayor, Campus Universitario, San Lorenzo 111241, Paraguay; 8 Department of Global Pediatric Medicine, St. Jude Children’s Research Hospital, Memphis, TN 38105, USA; hyunjy.chung@stjude.org; 9 Department of Infectious Diseases, St. Jude Children’s Research Hospital, Memphis, TN 38105, USA; richard.webby@stjude.org; 10 Department of Pediatrics, University of Tennessee Health Science Center College of Medicine, Memphis, TN 38103, USA

**Keywords:** COVID-19 laboratory, pandemic preparedness, low- and middle-income countries, research, Paraguay

## Abstract

Soon after the declaration of the COVID-19 pandemic, the Institute for Health Sciences Research (IICS) of the National University of Asunción, Paraguay became a testing laboratory (COVID-Lab) for SARS-CoV-2. The COVID-Lab testing performance was assessed from 1 April 2020 to 12 May 2021. The effect of the pandemic on the IICS and how the COVID-Lab contributed to the academic and research activities of the institute were also assessed. IICS researchers and staff adjusted their work schedules to support the COVID-Lab. Of the 13,082 nasopharyngeal/oropharyngeal swabs processed, 2704 (20.7%) tested positive for SARS-CoV-2 by RT-PCR. Of the individuals testing positive, 55.4% were female and 48.3% were aged 21–40 years. Challenges faced by the COVID-Lab were unstable reagent access and insufficient staff; shifting obligations regarding research, academic instruction, and grantsmanship; and the continuous demands from the public for information on COVID-19. The IICS provided essential testing and reported on the progress of the pandemic. IICS researchers gained better laboratory equipment and expertise in molecular SARS-CoV-2 testing but struggled to manage their conflicting educational and additional research obligations during the pandemic, which affected their productivity. Therefore, policies protecting the time and resources of the faculty and staff engaged in pandemic-related work or research are necessary components of healthcare emergency preparedness.

## 1. Introduction

SARS-CoV-2 emerged in Wuhan, China, in December 2019 [1,2], and the COVID-19 pandemic was declared shortly thereafter [3]. The severity of COVID-19 ranges from asymptomatic cases to critical and terminal cases [2]. Affected individuals may experience mild constitutional symptoms, such as high fever, cough, myalgia, fatigue, and disorders of smell and taste, or more severe respiratory symptoms, acute heart injury, and even death [2,4,5]. The manifestations are linked to the effect of the infection on tissues and organs, the immune response to the disease, and the consequences of these events, i.e., post-COVID-19 effects [6]. Initially, COVID-19 was diagnosed by using nucleic acid amplification tests (NAATs) [7] to detect the SARS-CoV-2 RNA. Later, other diagnostic methods were used for viral detection [8].

Paraguay, like the rest of the Americas, has been severely affected by COVID-19 [9]. Surveillance of SARS-CoV-2, a key element of pandemic preparedness, was implemented at the Instituto de Investigaciones en Ciencias de la Salud (IICS), which was posed for this task. One of the most important assets of IICS is its highly trained and capable researchers, versed in molecular techniques, and well-connected with their international peers. IICS, part of the local university, has been providing scientific, academic, and clinical services to the national public health systems since before the onset of the pandemic. This country with 7 million inhabitants is organized territorially and governmentally into 17 departments, of which the Central department is the most populous, with 2,243,792 inhabitants (approximately 30.5% of the total population of the country) [10]. In March 2020, Paraguay reported its first confirmed case of COVID-19 in a 32-year-old man from Ecuador. The second case was reported 3 days later in an individual from Argentina [11]. A strict, nationwide, mandatory lockdown was quickly implemented as a preventive suppressive strategy [12], but this lockdown mandate was loosened as the months passed. When COVID-19 vaccines became available, first-line health workers were the first to receive them. In addition, during the pandemic, contingency laboratories were created. Thus, we describe the establishment of a contingency laboratory by the IICS, a local research and educational facility, to respond to the demands of the COVID-19 pandemic in Paraguay, and report the results of the testing performed there, along with our related experiences applicable to creating future strategies for similar situations.

## 2. Materials and Methods

### 2.1. Contingency Laboratory of the IICS of the UNA

At the onset of the pandemic, the Instituto de Investigaciones en Ciencias de la Salud (Institute for Health Sciences Research) (IICS) of the National University of Asunción (UNA), Paraguay, established itself as the contingency technical laboratory for COVID-19 diagnosis (COVID-Lab). The results of tests performed at the COVID-Lab were provided daily to the Ministry of Health to track the progress of the pandemic. Pre-pandemic, the IICS provided scientific, academic, and clinical diagnostic services to the Paraguayan community and led biomedical research efforts in Paraguay. As a research institution, it participated in national, regional, and global collaborative biomedical studies [13]. With oversight by the National Agency for Evaluation and Accreditation of Higher Education [14], the IICS offered graduate (MSc and PhD) degrees and short courses for gaining or updating specific skills, including diagnostic methods used in virology, bacteriology, and parasitology. The IICS laboratories provided diagnostic services to the community, including those using molecular and other modern laboratory techniques and resources (PCR and its variations, hybridization with nonradioactive probes, restriction fragment–length polymorphism (RFLP), ELISA, flow cytometry, pathological anatomy, nuclear medicine, cytogenetics, clinical and endocrinological analysis, research animal facilities, immunofluorescence, diagnostic kit production, Fourier-transform infrared spectroscopy, etc.). The IICS assisted the National Public Health systems by providing skills and expertise, as well as the workforce for generating information about endemic, epidemic, and pandemic diseases.

### 2.2. COVID-19 Testing

During the pandemic, the COVID-Lab analyzed oropharyngeal and nasopharyngeal (OP/NP) swabs collected mainly from patients cared for at the Hospital de Clínicas, a 600-bed teaching hospital of the UNA School of Medicine, and from a surveillance collection station (testing site) established at the exit from the UNA campus. SARS-CoV-2 detection was provided free of charge. The catchment areas for testing included Asunción proper (population: 500,000) and the surrounding communities of the Central Department (population: 2,243,792) [10]. Early in the pandemic, the bulk of COVID-19 testing was performed at the laboratories of the Paraguay Ministry of Public Health and Social Welfare (henceforth, the Ministry of Health). However, the demand for testing led to the emergence of multiple public and private molecular testing sites, mainly in major urban areas (Table 1). Regardless of the testing site, all test results were reported to the Ministry of Health, which kept a careful record of the number of COVID-positive tests.

### 2.3. Public Health Prevention Strategies for COVID-19

A strict mandatory lockdown, starting in early March 2020, was quickly implemented for the general population, excluding essential workers [12]. Subsequently, from early May through early July 2020, a “smart quarantine”, based on WHO recommendations, was implemented, and the lockdown was gradually loosened in stages. Thus, every 3 weeks, more population categories resumed their normal work and activities. The first to return were industrial workers and individual delivery services; next came workers in commercial stores, corporate offices, and construction businesses, along with sporting and cultural events (without spectators). In the third phase, the permitted number of attendees at in-person events was increased; and in the fourth phase, bars and restaurants were allowed to reopen and there was a resumption of the full capacity of in-person events, hospitality in general, and other social activities, with the use of face masks remaining mandatory [15]. This last phase lasted until the initiation of vaccination of the general population of Paraguay in April 2021 [16].

### 2.4. Laboratory Methods and Supplies for SARS-CoV-2 Testing

Clinical sample processing, RNA extraction, and viral detection by RT-PCR followed standard procedures [17]. However, because of the shortage of laboratory reagents in Paraguay and worldwide, the COVID-Lab used any available kits. For extracting viral RNA, the following kits were used: the ReliaPrep Blood gDNA Miniprep System (Promega, Madison, WI, USA), the VIASURE RNA-DNA Extraction Kit (CerTest Biotec, Zaragoza, Spain), the PURO Virus RNA Kit (PB-L Productos Bio-Lógicos, OneLab, Buenos Aires, Argentina), the ReliaPrep Viral TNA Miniprep System (Promega), the NX-48S Viral NA Kit (Genolution, Seoul, Republic of Korea), and the PureDireX Virus Nucleic Acid Isolation Kit (Bio-Helix, Keelung City, Taiwan) (Table 2). Kits used for RT-PCR reactions were the NeoPlex COVID-19 Detection Kit (GeneMatrix, Seongnam, Republic of Korea) and PowerChek 2019-nCoV Real-time PCR Kit (Kogene Biotech, Seoul, Republic of Korea) (Table 3). Subsequently, the method for detecting gene E, as outlined by Corman et al. [18], was implemented. Confirmatory testing used the CDC procedures for detecting the N2 gene [19], using the following kits: the Takyon Dry One-Step RT Probe MasterMix No Rox (Eurogentec, Seraing, Belgium), the GoTaq Probe 1-Step RT-qPCR System (Promega), the QUANTABIO qScript XLT One-Step RT-qPCR ToughMix (Quantabio, Beverly, MA, USA), the iTaq Universal Probes One-Step Kit (Bio-Rad, Hercules, CA, USA), and the Luna Universal Probe One-Step RT-qPCR Kit (New England Biolabs, Ipswich, MA, USA). Before starting qPCR testing, laboratory validation was conducted by the COVID-Lab staff, using positive and negative samples provided by the Ministry of Health Central Laboratory. Laboratory techniques employed included quantitative real-time PCR (qPCR) (both SyBr Green and TaqMan assays), PCR-RFLP, conventional PCR, RT-PCR, hybridization, next-generation sequencing (NGS), and the use of recombinant proteins. Supplies used by the COVID-Lab included those provided by Jesse Waggoner of Emory University. The International Atomic Energy Agency (IAEA) and the Mérieux Foundation provided other essential laboratory supplies.

### 2.5. Sample and Data Collection and Analysis

The NP/OP swabs were obtained from individuals who were suspected of having COVID-19, had been exposed to a patient with COVID-19 within the first week of their illness, or had other reasons to be tested. The swabs were processed at the COVID-Lab. Using a case report form, patient data were obtained from the epidemiologic online database of the General Directorate of Health Surveillance of the Ministry of Health [20]. Captured data included basic demographics, reasons for testing, disease symptoms, and co-morbid conditions. An Excel 2016 database template was populated with the case reports. Subsequently, basic statistics and online statistical tools were used to analyze and report the results.

### 2.6. Definitions

The study definitions were as follows: a case with no epidemiologic link was one in which the person with COVID-19 had no history of contact with a SARS-CoV-2–positive individual and no travel history; a contact case was one in which the person had been in close contact (in a household or work setting) with or within 1.5 m of a person with a confirmed case of COVID-19 for longer than 10 min [21]. The cycle threshold (Ct) was the number of cycles needed for an amplicon to become detectable above the established background.

### 2.7. Ethics

The manuscript for this publication was approved by the ethical and scientific committees of the IICS-UNA under code M06/2021.

## 3. Results

### 3.1. The Contingency Laboratory of the IICS of the UNA (COVID-Lab)

From the onset of the pandemic and throughout its course, multiple IICS staff members were responsible for running the COVID-Lab. These individuals moved their routine work as researchers and educators to the newly established contingency laboratory. Led by the chief of the molecular biology and biotechnology laboratories, a team of up to 14 individuals processed test samples, two individuals managed the data, and one served as an information technology technician. The COVID-Lab studied the circulating SARS-CoV-2 from June 2020 to May 2021. The RT-qPCR protocols were originally developed “in house”, following the protocols of the Charité and the CDC. Before commencing routine testing, the lab standardized the protocol, adapting it to the equipment and reagents available on the local market. The referral of samples to the COVID-Lab was approved by the General Directorate of Health Surveillance (DGVS) of the MSPyBS, in collaboration with the Hospital de Clínicas, the teaching hospital of the School of Medicine of the UNA. Early in 2021, the lab began using methods developed and published by Yale University to detect variants of concern and collaborated with scientists at Emory University to improve the methodology for detecting variants of concern through directed RT-PCR. The challenges encountered and addressed were the lack of sensitivity to the specified targets and the contamination of commercially obtained primers. At the time of writing (December 2022), COVID-19 testing was listed on the menu of diagnostic tests offered at the IICS and was available at a minimal charge. In addition, the COVID-Lab personnel continued to provide testing as requested, supported by a team of 10 individuals, but the number of tests performed each week shrunk from 400 at the peak of the pandemic to fewer than 10.

### 3.2. COVID-Lab Testing Results

Between 1 April 2020 (the start of COVID-Lab operations) and 12 May 2021, 13,082 COVID-19 tests were performed, using nasal and NP/OP swabs. Of these tests, 2704 (20.7%) were positive for SARS-CoV-2 by real-time RT-PCR. The first positive result for SARS-CoV-2 obtained by the COVID-Lab was registered on 1 June 2020; the first cases were in 16 travelers from Brazil and 4 from Chile. Overall, the COVID-Lab test reports reflected the availability of supplies rather than the dynamic of COVID-19 in the community.

### 3.3. Descriptive Data for Individuals Testing Positive for SARS-CoV-2

**Figure 1:**
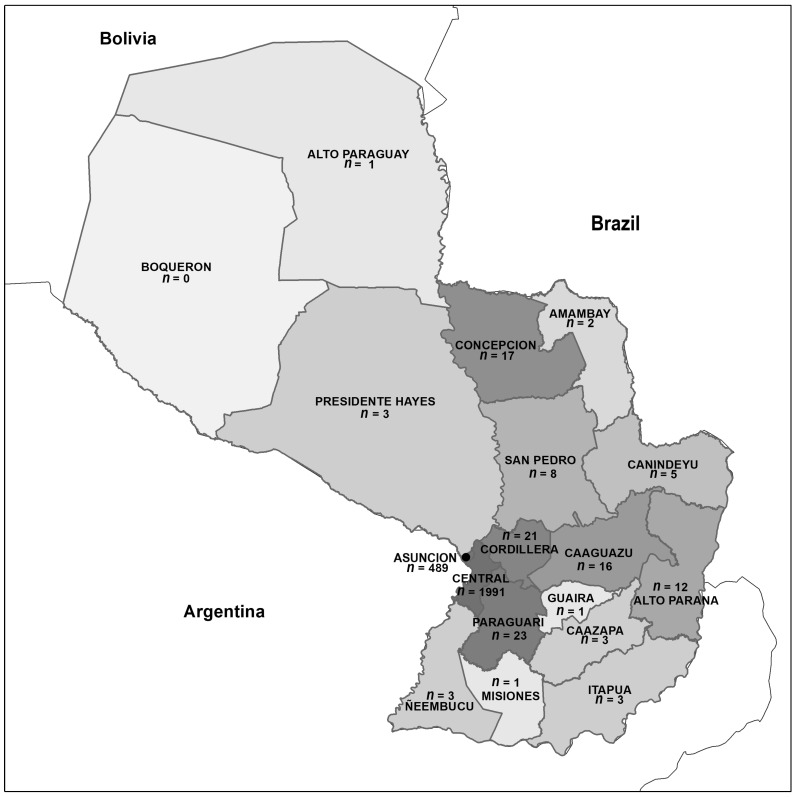
Origin of the individuals tested by the COVID-Lab who were found to be positive for SARS-CoV-2.

In our cohort of 2704 individuals who tested positive for SARS-CoV-2, 1498 (55.4%) were female and 2480 (91.7%) came from the central region of the country (489, 18.01% of the cohort, from Asunción proper and 1991, 73.6% of the cohort, from other cities of the Central department), with 119 (4.4%) of the patients coming from other regions (Figure 1 and Table 4).

SARS-CoV-2 was confirmed in individuals of all age groups (Table 5); however, the infection was most commonly detected in the groups aged 21–30 and 31–40 years, which accounted for 24% and 24.3% of the cases, respectively. Table 4 shows the distribution of the positive test results for SARS-CoV-2 by month and illustrates the challenges faced by the COVID-Lab during the pandemic. For 2 months (August and September 2020), there was no report made because of a scarcity of supplies. In December 2020 and January 2021, the number of positive cases increased because of processing samples collected at other sampling locations. However, the increase in positive cases in February and March 2021 coincided with the increased COVID-19 activity in the community [22].

### 3.4. Epidemiologic and Clinical Characteristics, Report Dynamic

The records for 455 of the 2704 patients in our cohort (16.8% of the total) included no information on their symptoms or lack thereof, whereas 1801 patients (66.6%) were reported to have at least one symptom and 448 (16.6%) were reported to be asymptomatic (Table 6 and Table 7).

For 276 of the 448 asymptomatic patients (61.6%), no specific reason for being tested was recorded. The remaining 172 asymptomatic patients were tested because they had had contact with a confirmed COVID-19 case (108 instances, 24.1% of the total), they were healthcare workers who required a routine test (27 instances, 6%), they required a pre-surgical test (27 instances, 6%), or they required a test upon returning from an endemic area (10 instances, 2.2%).

In the 1801 symptomatic cases, the most frequently reported clinical manifestations were pharyngitis (855 cases, 47.5%), cough (855 cases, 47.5%), headache (809 cases, 44.9%), referred fever (766 cases, 42.5%), nasal congestion (638 cases, 35.4%), dysgeusia (550 cases, 30.5%), anosmia (539 cases, 29.9%), and myalgia (360 cases, 20%). Other common complications were shortness of breath (338 cases, 18.8%), coryza or rhinorrhea (277 cases, 15.4%), and gastrointestinal complications (Table 7).

### 3.5. Co-Morbidities and Underlying Health Problems

The most common risk factors identified in the 242 individuals who tested positive for SARS-CoV-2 by RT-PCR were diabetes (93 individuals, 38.4%), chronic heart disease (49, 20.2%), asthma (41, 16.9%), obesity (43, 17.8%), and chronic kidney disease (38, 15.7%) (Table 6). In this cohort, 238 individuals had more than one co-morbid condition, including diabetes with chronic heart disease (15 individuals, 6.3%), diabetes with chronic kidney disease (12 individuals, 5%), and chronic heart disease with chronic kidney disease (11 individuals, 4.6%).

### 3.6. Impact of COVID-19 on the IICS

The IICS was affected by the COVID-19 pandemic in the areas of education, research, and diagnostic services. The teachers and members of the educational department strove to continue imparting training despite their new roles in the COVID-Lab. Students and their workstations were relocated outside the COVID-Lab to enable them to continue working on their projects. Some students participated in specific areas of work in the COVID-Lab, adhering to the required infection prevention and control procedures. With respect to research, the molecular biology department benefitted most from the pandemic by obtaining better infrastructure for operating their laboratories, including laboratory equipment received as international donations. Furthermore, a portable sequencer was acquired through a research project funded by an express call by the government research granting agency CONACYT (Consejo Nacional de Ciencia y Tecnología) to support COVID-19 research. In response to this call, the local granting agency covered all expenses of the portable sequencer and the supplies in record time, bypassing multiple routine bureaucratic steps. Other research departments derived less benefit for various reasons. Because IICS is a research institute, the research teams were assigned specific days for coming to the institute to work on their projects. Inevitably, much of the research was stalled, as were procuring grants and generating papers to disseminate the results of the work. These delays may have a negative impact on the future financial support from the national programs in which the researchers are enrolled, as academic and scientific productivity are among the metrics used to qualify for such support. As for the staff of the COVID-Lab, those researchers devoted their time and expertise to solving the problems raised by the pandemic. Government agency-financed projects focused on COVID-19 rather than on other lines of research. Regarding diagnostic services, the COVID-Lab was incorporated as a specialty laboratory, and testing for SARS-CoV-2 was added to the menu of available diagnostics.

## 4. Discussion

We have reported our experience of establishing and operating a contingency laboratory for COVID-19 at a research and educational institution in Paraguay. Despite challenges in accessing supplies and adjusting the routine pre-pandemic educational and research responsibilities of the staff, the laboratory became the main provider of testing for a teaching hospital that cares for the sickest and poorest patients in the population. The results reported after 1 year reflect the characteristics of the COVID-19 pandemic in the population tested in Paraguay before the initiation of vaccination. The study found that respiratory samples from 20.7% of the individuals requiring a COVID-19 test were positive for the virus. Approximately half of these individuals were 20–40 years of age, and more than two-thirds were symptomatic, with constitutional symptoms being the most frequent. Diabetes and chronic cardiopathy were frequent co-morbidities in the tested cohort. Laboratory supplies originating from multiple manufacturers were used for the molecular tests for SARS-CoV-2, reflecting the overall performance of industries supporting diagnostics during the pandemic.

Shortly after the onset of the pandemic, the IICS established a COVID-19 testing site on the UNA campus in the San Lorenzo suburb of Asunción. The initial testing for SARS-CoV-2 was based on molecular techniques. The familiarity of the IICS staff with such techniques and the availability of the necessary equipment at the institution enabled COVID-19 diagnostic testing to be implemented successfully. However, access to supplies was an important challenge. The standard methods used for COVID-19 diagnostics are molecular, antigen detection, and serologic techniques [23]. Each of these methods has advantages and disadvantages. Molecular testing uses PCR technology and is the gold standard for COVID-19 testing, enabling both the presence of the viral RNA and the viral load to be determined. Similarly, antigen testing relies on the presence of viral antigens in the sample and is less sensitive than the PCR-based methods [8]. A serologic test depends on the presence of anti–SARS-CoV-2 antibodies in the serum, and this is usually detected later in the infection course or after the infection has resolved [24]. In many low- and middle-income countries (LMICs), barriers to COVID-19 testing include testing costs, supply chain constraints, a lack of uniform information about testing, and the associated stigma and potential consequences for individuals who have positive test results [25]. Organizations such as the World Health Organization, UNICEF, and the Rockefeller Foundation are working with multiple partners, including industry representatives, to provide guidance on using and upscaling the available COVID-19 diagnostics, as well as those still under development, in LMICs [26,27,28]. Reducing the barriers to testing by augmenting global resources for PCR, antigen testing, and serology, especially in LMICs, is essential to controlling the pandemic and to decreasing the health gaps around the globe. In our institution, the IICS, the pandemic intensified the use of molecular techniques, including the introduction of the use of next-generation sequencing (NGS). Furthermore, these laboratory innovations have found applications in the study of other important pathogens for the country and region.

As noted in Section 3.3. the COVID-Lab had to suspend testing for 2 months because of a lack of supplies at a time when the number of cases in Paraguay and neighboring countries was rising [23]; however, testing continued in other laboratories in the country. Latin America faced the pandemic in a state characterized by suboptimal healthcare investment and pandemic preparedness, which resulted in the poor performance by the various national coordinated testing programs [29]. Early in the pandemic, the Pan American Health Organization collaborated with public health laboratories in Latin American countries, including Paraguay, to implement the molecular detection of SARS-CoV-2 [30]. However, despite well-organized regional and national implementation plans, access to reagents was uneven at the IICS and access to testing services was unavailable in less urbanized areas and rural areas, most likely resulting in a significant underreporting of cases in Paraguay. In other countries with similar economic backgrounds, such as Rwanda, a coordinated response was mounted that included steady access to testing [31]. In this Central African country, early in the pandemic, healthcare leaders understood the crucial role of testing, and the government invested in sufficient equipment, supplies, and processes to facilitate the analysis of results at a central site. A coordinated national response to a health emergency requires bringing together a multitude of stakeholders and sectors to ensure a robust solution. Early in the pandemic, the WHO outlined the essential elements of preparedness, which included national preparedness and response operations, in which testing was central for the rapid identification, diagnosis, and management of cases and the follow-up of contacts [32]. During a health crisis, a coordinated national plan for health and support efforts, including testing and surveillance, should be operational to avoid extended shortages of supplies in laboratories that serve an important and vulnerable population. For a national contingency plan for responding to COVID-19 to be effective, robust preparedness should include obtaining the wide participation of existing expertise, strengthening and supporting reputable laboratories, such as the IICS COVID-Lab, and equipping such facilities with sufficient resources to handle the cyclic surges during the pandemic [33].

The researchers and educators and their staff at the IICS supported the contingency laboratory during the peak and subsequent waves of the pandemic. In Paraguay, the IICS is the main biomedical research institution and is dependent on the UNA. For the researchers at the institution to maintain their certification as researchers and qualify to apply for grants, they must comply with the specific requirements dictated by the government research granting agency, CONACYT. In Paraguay, researchers are systematically ranked by CONACYT, and only if they are found qualified can they apply for grants. The rankings are based on the research projects conducted and the publications produced by the researchers that pertain to their field of interest. During the pandemic, the COVID-Lab team was a multidisciplinary group led by a few experts in virology who worked and generated news and publications on COVID-19 [34]. The collaboration of professionals and academic institutions with expertise in molecular biology, beyond the strictly biomedical sciences, was common in the region, such as in Argentina and Uruguay [35]. The broad community of molecular biologists and other experts outside the biomedical science field were able to develop testing procedures and, thus, enable easier access to testing and even the identification of variants [34]. Often, however, the presence of technical expertise during a pandemic is not enough; there is a need for steady access to critical laboratory supplies and centralized coordination to ensure a consistent response by testing sites such as the IICS COVID-Lab. Country-wide and institutional policies, which explicitly prioritize the use of existing expertise and resources during the pandemic, must be in place to guide personnel allocations, job descriptions, and deliverables, including the production of some supplies locally. Such policies should also provide guidance to researchers and educators during a health emergency. The guidance should cover the continued performance of routine research and/or educational activities and criteria for re-appointments and promotions relevant to those engaged in pandemic-related work, as well as to those whose jobs have been put on hold because of the pandemic. Importantly, the response to the COVID-19 pandemic and the testing workforce is a marker of quality; however, for a prolonged pandemic of 2–3 years, there must be an explicit policy to guide the post-pandemic reintegration of personnel and to provide the appropriate recognition and consideration of those who postponed their research and projects to participate in the COVID-Lab. Therefore, investing in and protecting human expertise and availability should be a priority in a pandemic preparedness plan.

As the COVID-Lab served mainly the teaching hospital, which cares for the poorest and sickest people, it is not surprising that a high percentage of those who tested positive for SARS-CoV-2 had co-morbidities. The most frequent co-morbidity was diabetes, followed by chronic heart disease. Often, these and other underlying conditions were associated with each other; for example, diabetes with chronic heart disease, diabetes with chronic kidney disease, and chronic heart disease with chronic kidney disease. Consistent with the published literature, variables affecting the severity of COVID-19 were the age of the patient and chronic health conditions pre-existing the SARS-CoV-2 infection [36]. These data inform us about the degree of susceptibility of the IICS-tested population, especially the older patients and those with underlying diseases, who were prone to developing more severe forms of COVID-19, including respiratory distress syndrome, multiple organ failure, and death [37]. In contrast, young adults most frequently had no co-morbidities. In our cohort, 10.4% of the individuals who tested positive for SARS-CoV-2 were healthcare personnel, including both symptomatic and asymptomatic cases. Healthcare workers are at an increased risk for acquiring COVID-19, possibly because of increased exposure and suboptimal adherence to infection prevention and control practices. Importantly, once community transmission is established, healthcare personnel can be exposed at work and in the community. According to a prospective cohort study, frontline healthcare workers had an increased risk of COVID-19 infection influenced by suboptimal infection control practices [38]. However, another study indicated that the rates of infection and mortality from COVID-19 among healthcare workers mirrored those of the general population [39]. The strategic location of testing sites maximized the benefits to the population, as was the case with the IICS COVID-Lab, which was at one of the largest hospitals serving the poor and uninsured population. In addition, the contingency laboratories were easily accessible to the community surrounding the UNA campus, which is one of the largest in the country, with approximately 50,000 students and more than 9000 faculty [40]. Therefore, it was not surprising that the central region had the highest number of positive-testing individuals.

As a neighbor of Brazil and Argentina, Paraguay enjoys multiple benefits of being bordered by more economically affluent countries but must also contend with the multitude of associated ills, especially the effect of Punt Politics in Brazil during the pandemic [41]. At the onset of the pandemic, Paraguay was the first nation in the region to implement a country-wide lockdown [42] and was successful in slowing COVID-19 transmission. However, this pandemic control strategy was impossible to sustain for several reasons. First, as in other Latin American countries [29], nearly 70% of the total employment in Paraguay was informal [43], characterized by instability, lower income, and a lack of health benefits. Second, the MERCOSUR trade agreement between Argentina, Brazil, Paraguay, and Uruguay had given rise to a micro-economy based on border crossing by workers and traders, often daily, and especially from Brazil and Argentina. Third, the dry borders, with multiple crossing points and no geographical barriers, facilitated a migratory flow. Despite the regulations in place [16,42], it was impossible to enforce and control the movement of people across borders, and the suppression strategy to prevent COVID-19 transmission was impossible to sustain for this type of population. In addition, the management of the pandemic in bordering countries, e.g., Brazil, which allowed unrestricted movement within the country and across its borders, provided the gateway by which SARS-CoV-2 entered Paraguay, causing an outbreak that was challenging to contain and control [44]. Vargas-Correa et al. (2021) reported a prevalence of 36.1% positivity for SARS-CoV-2 (1754 of 4855 individuals tested), as detected by molecular methods, between 12 August and 20 October 2020, with most cases being located in the department of Alto Paraná, which borders Brazil [45]. Paraguay experienced the brunt of the pandemic for various reasons: first, because of the suboptimal national healthcare situation when the pandemic spread throughout the country; second, by being vulnerable to the pandemic-related havoc in neighboring countries; third, by the politicization of the pandemic and misinformation exacerbating transmission; and fourth, by being outcompeted for limited resources such as vaccines and other COVID-19 therapeutics by other countries. However, by tapping into the existing resources, the goodwill of donors, and government investment in healthcare infrastructure, testing was put into place, the number of critical care beds at the teaching hospital was increased from 17 (pre-pandemic) to 25 (in mid-2021) [46], and the local CONACYT provided the equipment necessary to study the variants circulating in the country. Additionally, the country-wide lockdown contained the spread of COVID-19 in the early months of the pandemic, giving time to increase healthcare resources and develop contingency health policies. However, compliance with this lockdown was challenging because of the socioeconomic disruption of the Paraguayan population and the unsustainable dynamic of people crossing the borders from surrounding countries where the pandemic was already severe. The magnitude of the task of implementing and sustaining lockdowns was underestimated; in the future, the practicalities of such interventions and the regional population movement dynamic must be taken into consideration to prevent or mitigate the socioeconomic disruption caused by a lockdown.

Our work is significant not only for the ongoing evolution of SARS-CoV-2 and the emergence of mutants but also for its use in other important endemic, epidemic, and emerging infections. [47] Prior to the implementation of genomic surveillance in the country, we already were detecting variants using published methods (Yale and Emory methods (RT-PCR)) and reporting our results of the identified variants entering the country to the Ministry of Public Health and Social Welfare. With the expertise gained, the equipment obtained, and a dynamic collaboration with our international peers, our COVID-Lab continues to study the evolution and emergence of mutants and keep abreast of the latest development in the field. Furthermore, the progress of this lab qualifies IICS to acquire and incorporate more sophisticated equipment into the infrastructure for use in other studies on a permanent, rather than contingent, basis. This will enable studies not only of SARS-CoV-2 but also of other pathogens of interest in public health (e.g., dengue, chikungunya, mycobacteria, etc.), to which we can apply our expertise developed with SARS-CoV-2. In addition, we have access to respiratory samples for COVID-19 testing from a high-risk population via the Hospital de Clinicas, a national referral center for serious and routine diseases in adults and children, especially for disadvantaged populations, and this will assist us with monitoring the circulating SARS-CoV-2 strains to detect the appearance of new variants and other emerging pathogens. Finally, the pandemic has resulted in the establishment and expansion of a cadre of virologists, public health experts, educators, and in-training individuals at the IICS, considerably enhancing our ability to respond to the emergence of new variants of SARS-CoV-2 and other pathogens.

## 5. Limitations

A main limitation of our study is the incomplete information on the individuals who were tested for COVID-19, including data related to mortality. This limitation is to be expected, given that our work is a retrospective study. However, because of the large number of individuals tested for COVID-19, important information was nevertheless obtained. Another limitation of our study is the ongoing development of our COVID-Lab, which has since evolved from a contingency laboratory to a molecular testing facility that incorporates new technology such as next-generation sequencing to the study of other important public health-relevant pathogens.

## 6. Conclusions

In conclusion, we reviewed the 1-year experience of a contingency laboratory in Paraguay that was established using the available biomedical research and educational infrastructure and expertise. A total of 20.7% of the suspected cases of COVID-19 were confirmed. Despite the quality of the testing offered and the critical population served by the testing center, the smooth functioning of the testing was affected by inconsistencies in access to laboratory supplies and suboptimal country-wide collaboration, the need to share laboratory supplies, and communication deficits. Importantly, researchers who participated in the contingency laboratory and those who could not participate were also affected by the pandemic. The local research structure was not adequately prepared to tolerate and grant concessions to researchers who could not sustain the expected pace of research during the pandemic because they were working in the contingency laboratory or because their work was restricted because of the pandemic. Paraguay was severely affected by the internal pandemic management and that of surrounding countries. While COVID-19 had a profound impact on the overall country response, especially on the IICS, this experience is not unique. Other countries also had challenges related to cultural norms and healthcare resources [48]. In a health emergency, adhering to country-wide collaboration and communication policies is important; even more so are policies on post-pandemic adjustment, which should give consideration to those affected by the pandemic so that they can return to normalcy.

## Figures and Tables

**Table 1:** Summary of the public and private laboratories authorized by the Ministry of Health to test for SARS-CoV-2 by molecular and immunochromatographic methods [1,2].

Summary of Laboratories Performing PCR Tests
13 public laboratories
2 centers with agreements with the Ministry of Health
2 self-sufficient institutions (the IICS and a Paraguayan military institution)
23 private laboratories
**Summary of private laboratories performing immunochromatography**
140 private laboratories enabled to perform antigen detection tests

**Table 2:** Viral RNA extraction supplies, their manufacturers and prices, and the total number of units used.

Reaction Kit	Manufacturer, Country	Validation Testing of Diagnostic Kits at the IICS ^1^	Price per Kit in USD *	Total Number of Kits Used
ReliaPrep Blood gDNA Miniprep System https://worldwide.promega.com/products/nucleic-acid-extraction/genomic-dna/reliaprep-blood-gdna-miniprep-system/?catNum=A5081 (accessed on 7 April 2023)	Promega, Madison, WI, USA	Yes	1621.52	4 kits, with 250 individual testing reactions in each kit Total individual testing reactions: 1000
VIASURE RNA-DNA Extraction Kit https://www.certest.es/wp-content/uploads/2019/02/VIASURE_EN_Extraction-KIT.pdf (accessed on 7 April 2023)	CerTest Biotec, Zaragoza, Spain	Yes	280.52	5 kits, with 50 individual testing reactions in each kit Total individual testing reactions: 250
PURO Virus RNA Kit https://www.onelab.com.ar/kit-para-purificacion-viral-puro-virus-rna (accessed on 7 April 2023)	PB-L Productos Bio-Lógicos, OneLab, Buenos Aires, Argentina	Yes	Donated	2 kits, with 100 individual testing reactions in each kit. Total individual testing reactions: 200
ReliaPrep Viral TNA Miniprep System https://worldwide.promega.com/products/nucleic-acid-extraction/viral-rna-extraction-viral-dna-extraction/reliaprep-viral-tna-miniprep-system-custom/?catNum=AX4820 (accessed on 7 April 2023)	Promega, Madison, WI, USA	Yes	1621.52	2 kits, with 250 individual testing reactions in each kit Total individual testing reactions: 500
NX-48S Viral NA Kit http://genolution.co.kr/product/dna-rna-extraction-kit/ (accessed on 7 April 2023)	Genolution, Seoul, Republic of Korea	Yes	708.70	10 kits, with 96 individual testing reactions in each kit Total individual testing reactions: 960
PureDireX Virus Nucleic Acid Isolation Kit http://www.bio-helix.com/products/184 (accessed on 7 April 2023)	Bio-Helix, Keelung City Taiwan	Yes	304.15	30 kits, with 100 individual testing reactions in each kit Total individual testing reactions: 3000

* Prices given are US dollar equivalents of the price in Guaranies (the Paraguayan currency) during the study period (1 April 2020 to 1 May 2021). ^1^ Diagnostic kits must be validated to demonstrate that they are scientifically sound, reliable, reproducible, and fit for their intended use. Each time a new kit was opened, a SARS-CoV-2–positive sample with a low Ct value, from the COVID-Lab stock, was used as a positive control.

**Table 3:** Rt-PCR reaction laboratory supplies, their manufacturers, and the total number used.

Reaction Kit	Manufacturer, Country	Validation Testing of Diagnostic Kits at IICS ^1^	Price per Kit in USD *	Total Number of Kits Used
GeneMatrix NeoPlex COVID-19 Detection Kit	Daewangpangyo-ro, Republic of Korea	Yes ^#^	Donated	2 kits, with 100 individual testing reactions in each kit Total individual testing reactions: 200
Kogene Biotech PowerChek 2019-nCoV Real-Time PCR Kit	Seoul, Republic of Korea	Yes ^#^	Donated	10 kits, with 100 individual testing reactions in each kit Total individual testing reactions: 1000
Eurogentec Takyon Dry One-Step RT Probe MasterMix No Rox	Seraing, Belgium	Yes ^#^	Donated	Total individual testing reactions: 1250
Promega GoTaq Probe 1-Step RT-qPCR System	Madison, WI, USA	Yes ^#^	501.99	3 kits, with 200 individual testing reactions in each kit Total individual testing reactions: 600
QUANTABIO qScript XLT One-Step RT-qPCR ToughMix	Beverly, MA, USA	Yes ^#^	538.13	6 kits, with 500 individual testing reactions in each kit Total individual testing reactions: 3000

* Cash donations in Paraguayan Guaranies and Euros have been converted to USD. Prices given are US dollar equivalents of the price in Guaranies or Euros during the study period (1 April 2020 to 1 May 2021). ^#^ The control sample of the PowerChek 2019-nCoV Real-Time PCR Kit (Kogene Biotech, Seoul, Republic of Korea) was used as a positive control. ^1^ Diagnostic kits must be validated to demonstrate that they are scientifically sound, reliable, reproducible, and fit for their intended use. Assay validation studies should be conducted to demonstrate the kit’s diagnostic accuracy, analytical sensitivity, analytical specificity, and ruggedness.

**Table 4:** Positive SARS-CoV-2 tests by RT-PCR at the COVID-Lab by department and by month in 2020 and 2021.

Department	2020							2021					Total *n* (%)
	June	July	August *	September *	October	November	December	January	February	March	April	May	
Alto Paraguay											1		1 (0.04)
Alto Paraná					1		1	2	2	2	2	2	12 (0.44)
Amambay										1		1	2 (0.07)
Asunción	3	2			18	11	103	21	31	224	48	28	489 (18.08)
Caaguazú	8					1	2	1	1	2	1		16 (0.59)
Caazapá									1		1	1	3 (0.11)
Canindeyú					1		2		1			1	5 (0.18)
Central	8	6			75	86	830	75	178	380	184	169	1991 (73.63)
Concepción		11			1	1	1		1	2			17 (0.63)
Cordillera					2	2	1	3	3	4	3	3	21 (0.78)
Guairá												1	1 (0.04)
Itapúa					1					1		1	3 (0.11)
Misiones					1								1 (0.04)
Ñeembucú						1		1	1				3 (0.11)
Paraguarí	1				2	1	4		10	3	2		23 (0.85)
Presidente Hayes						1	1				1		3 (0.11)
San Pedro	3				2	1	1		1				8 (0.3)
No data		1			4	8	51	4	6	21	2	8	105 (0.88)
Total	23	20			108	113	997	107	236	640	245	215	2704

* No supplies were available during these months.

**Table 5:** Positive SARS-CoV-2 tests by RT-PCR at the COVID-Lab by age group.

Age (Years)	*n*	%	Male	%	Female	%
0–10	45	1.7	16	1.3	29	1.9
11–20	183	6.8	95	7.9	88	5.9
21–30	650	24.0	263	21.8	387	25.8
31–40	658	24.3	313	26.0	345	23.0
41–50	409	15.1	181	15.0	228	15.2
51–60	337	12.5	135	11.2	202	13.5
61–70	196	7.2	90	7.5	106	7.1
71–80	93	3.4	51	4.2	42	2.8
81–90	30	1.1	13	1.1	17	1.1
91–100	6	0.2	1	0.1	5	0.3
No data	97	3.6	48	4.0	49	3.3
Total	2704	100	1206	100	1498	100

**Table 6:** Positive SARS-CoV-2 tests by RT-PCR at the COVID-Lab by sex and risk factors.

Risk Factors	*n* = 242	%	Female *n* = 143	%	Male *n* = 99	%
Diabetes	93	38.4	60	42.0	33	33.3
Chronic heart disease	49	20.2	28	19.6	21	21.2
Asthma	41	16.9	29	20.3	12	12.1
Obesity	43	17.8	27	18.9	16	16.2
Chronic kidney disease	38	15.7	17	11.9	21	21.2
Immunodeficiency disease/treatment	15	6.2	8	5.6	7	7.1
Chronic pulmonary disease	14	5.8	9	6.3	5	5.1
Arterial hypertension	12	5.0	7	4.9	5	5.1
Chronic neurologic disease	8	3.3	4	2.8	4	4.0
Chronic liver disease	6	2.5	0	0.0	6	6.1
Down syndrome	1	0.4	1	0.7	0	0.0
Malnutrition	0	0.0	0	0.0	0	0.0

**Table 7:** Positive SARS-CoV-2 tests by RT-PCR at the COVID-19-Lab by symptoms.

Total SARS-CoV-2 Positive	Total = 2704		%
Symptom	*n* = 1801	(%)	66.6
Pharyngitis	855	(47.5)	
Cough	855	(47.5)	
Headache	809	(44.9)	
Referred fever	766	(42.5)	
Nasal congestion	638	(35.4)	
Dysgeusia	550	(30.5)	
Anosmia	539	(29.9)	
Myalgia	360	(20.0)	
Shortness of breath	338	(18.8)	
Coryza or rhinorrhea	277	(15.4)	
Temperature > 38 °C	227	(12.6)	
Diarrhea	204	(11.3)	
Nausea or vomiting	165	(9.2)	
Abdominal pain	144	(8.0)	
Tachypnea dyspnea	60	(3.3)	
Prostration	57	(3.2)	
Abnormal lung auscultation	51	(2.8)	
Earache	42	(2.3)	
Irritability/confusion	37	(2.1)	
Conjunctival injection	24	(1.3)	
Seizures	4	(0.2)	
**No symptoms**	***n* = 448**		16.6
**No data**	***n* = 455**		16.8
**Total SARS-CoV-2 positive**	**2704**		**100**

## Data Availability

The data used in the current study are available from the corresponding authors M.A.C. and E.N. upon reasonable request.
